# Supplementary figures and images for: SMARCAD1 and TOPBP1 contribute to heterochromatin maintenance at the transition from the 2C-like to the pluripotent state
Source: eLife. 2025 Feb 19;12:RP87742. doi: 10.7554/eLife.87742 (PMC11839162; doi:10.7554/eLife.87742)

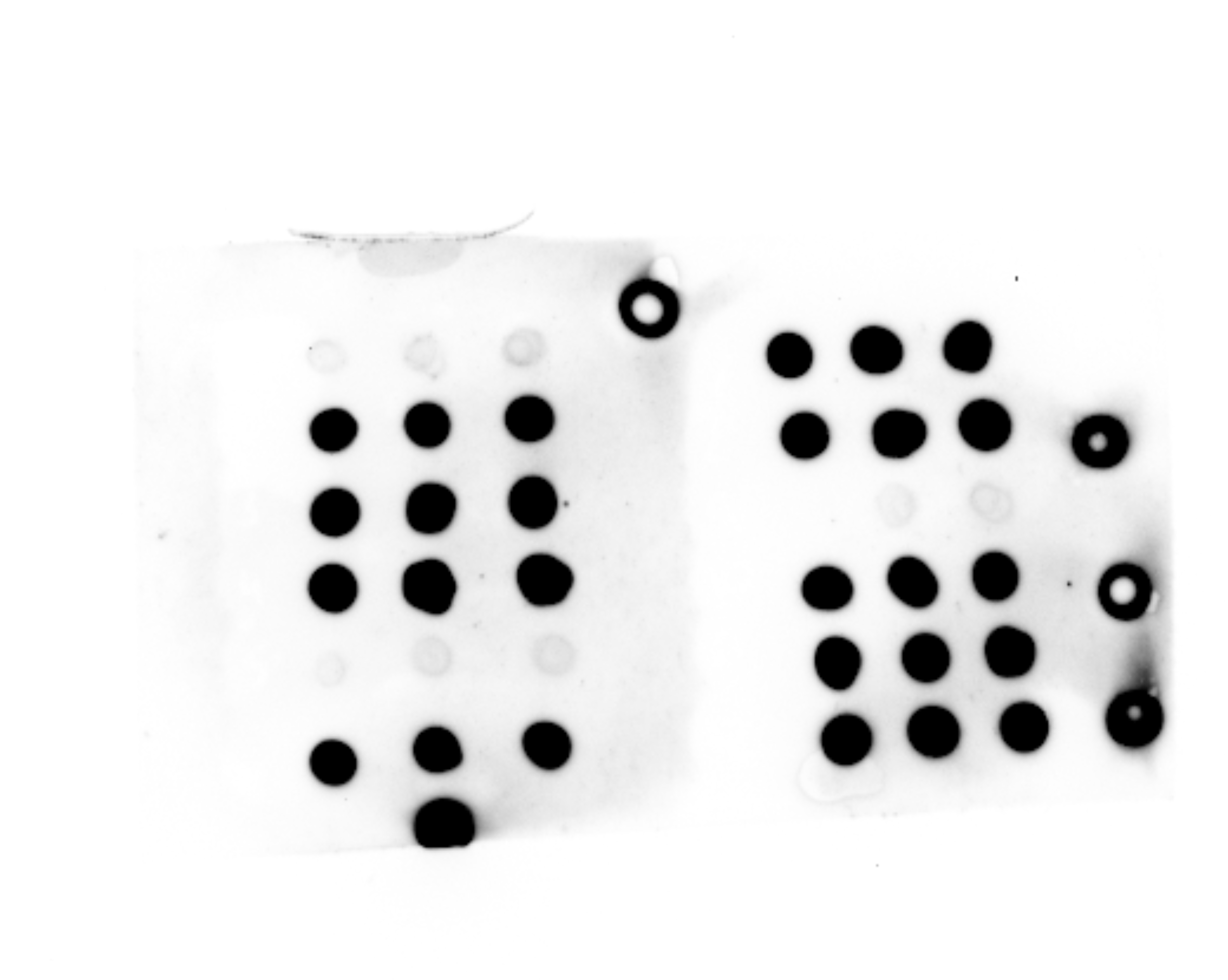

Supplement: Figure 2—figure supplement 1—source data 1. [file elife-87742-fig2-figsupp1-data1.zip › Figure 2-figure supplement 1_Source data 1/Dotblot_anti_H3.tif]

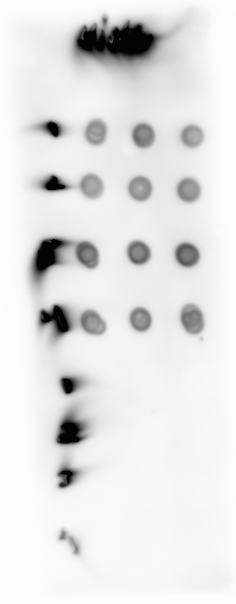

Supplement: Figure 2—figure supplement 1—source data 1. [file elife-87742-fig2-figsupp1-data1.zip › Figure 2-figure supplement 1_Source data 1/Dotblot_anti_Vinculin.tif]

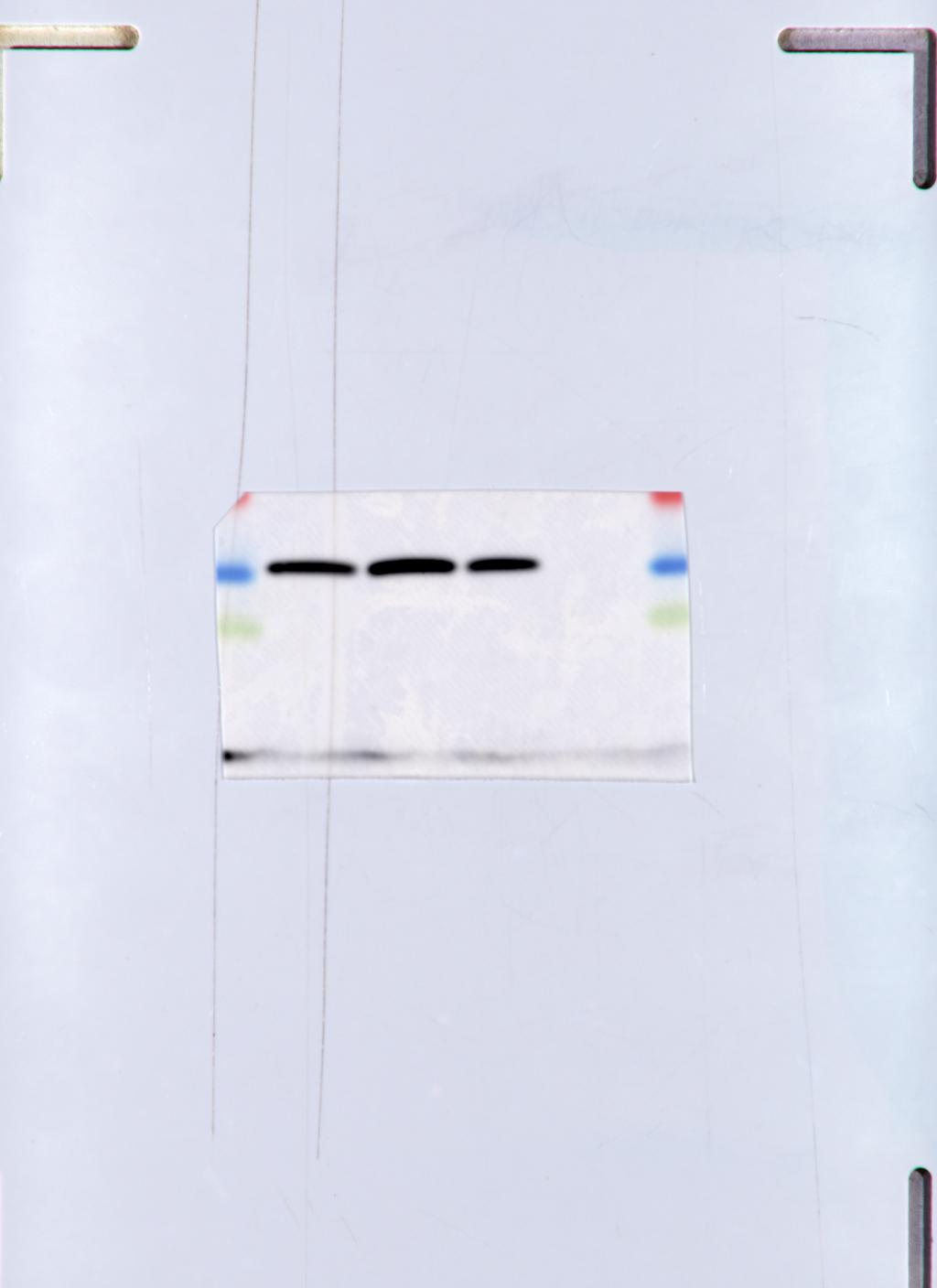

Supplement: Figure 3—figure supplement 1—source data 1. [file elife-87742-fig3-figsupp1-data1.zip › Figure 3-figure supplement 1_Source data 1/WB_anti_H3K9me3.tif]

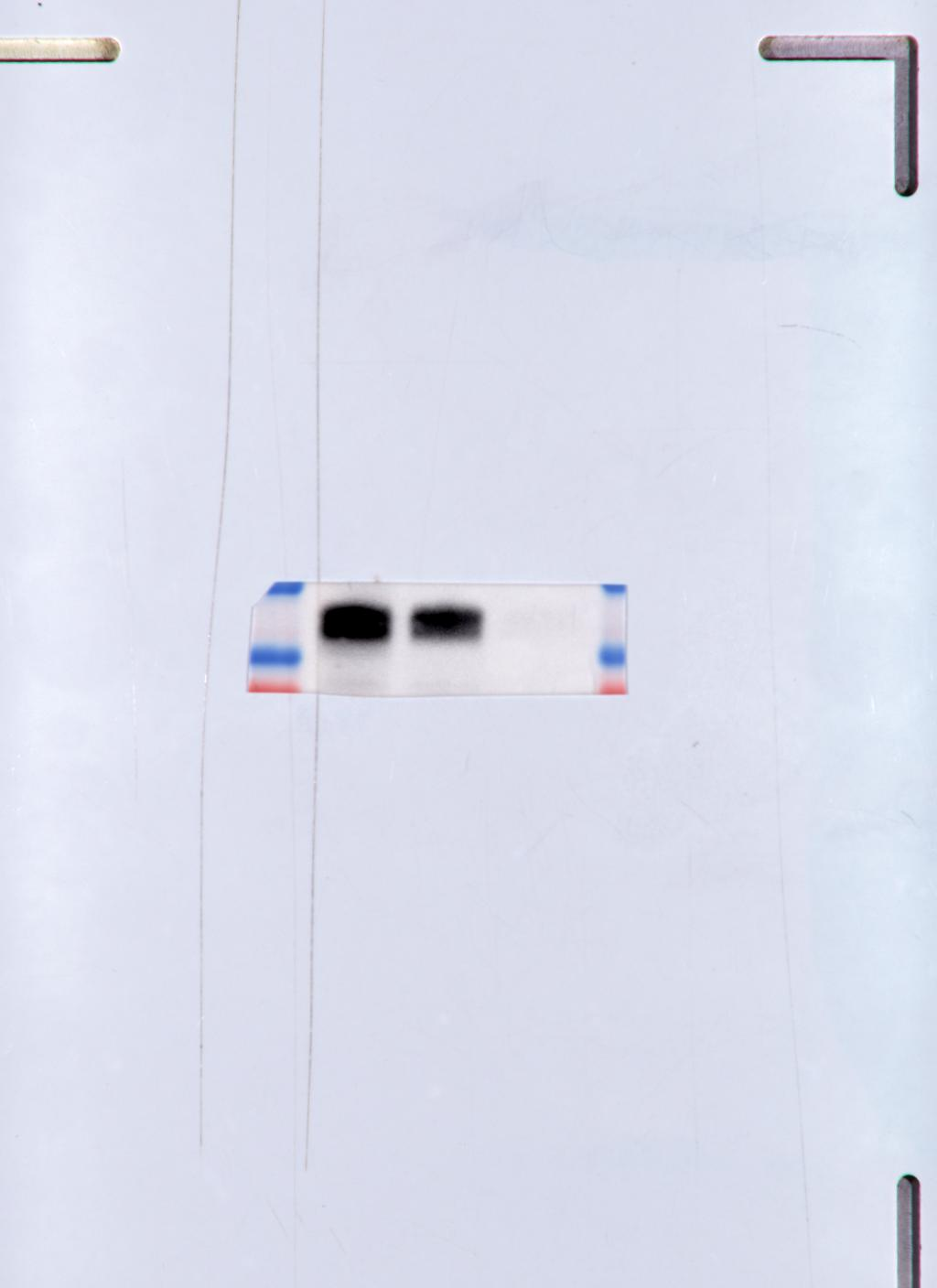

Supplement: Figure 3—figure supplement 1—source data 1. [file elife-87742-fig3-figsupp1-data1.zip › Figure 3-figure supplement 1_Source data 1/WB_anti_OCT4.tif]

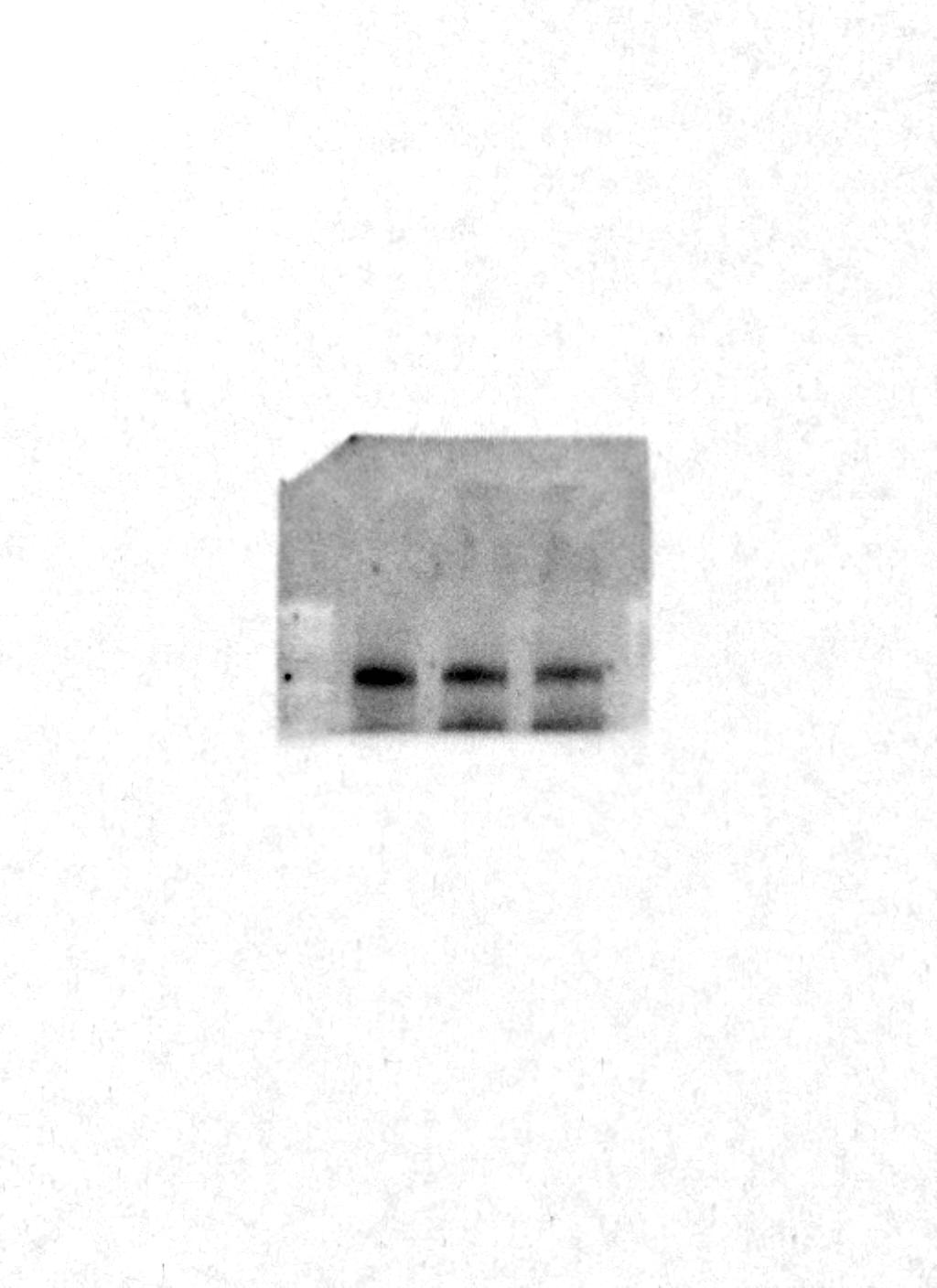

Supplement: Figure 3—figure supplement 1—source data 1. [file elife-87742-fig3-figsupp1-data1.zip › Figure 3-figure supplement 1_Source data 1/WB_anti_SMARCAD1.tif]

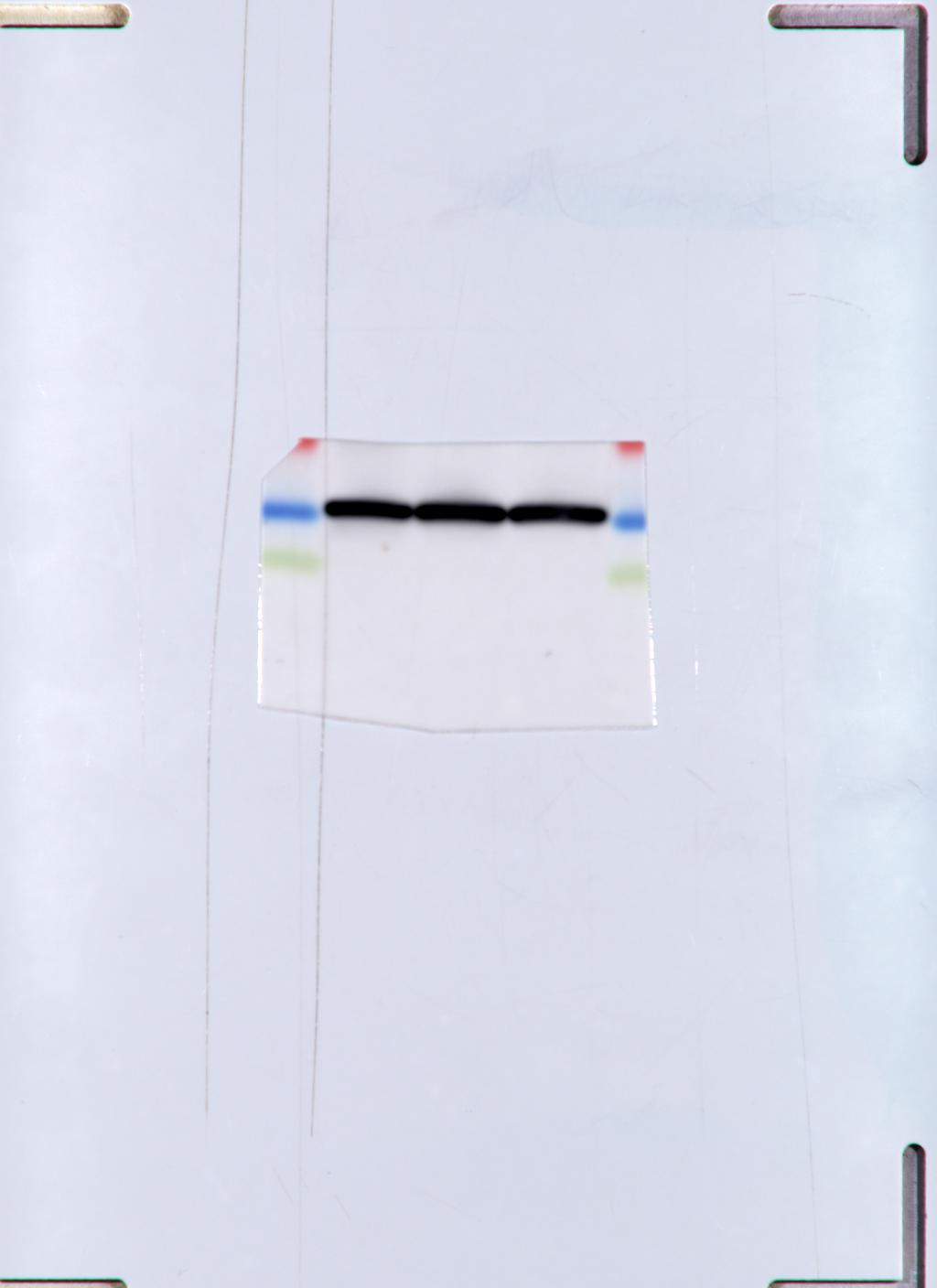

Supplement: Figure 3—figure supplement 1—source data 1. [file elife-87742-fig3-figsupp1-data1.zip › Figure 3-figure supplement 1_Source data 1/WB_anti_Total H3.tif]
