# Supplementary material for: SMARCAD1 and TOPBP1 contribute to heterochromatin maintenance at the transition from the 2C-like to the pluripotent state
Source: eLife. 2025 Feb 19;12:RP87742. doi: 10.7554/eLife.87742 (PMC11839162; doi:10.7554/eLife.87742)
Supplement: Figure 2—figure supplement 1—source data 2. [file elife-87742-fig2-figsupp1-data2.zip › Figure 2-figure supplement 1_Source data 2/Figure 2-figure supplement 1_Source data 2.pdf]

Anti-H3

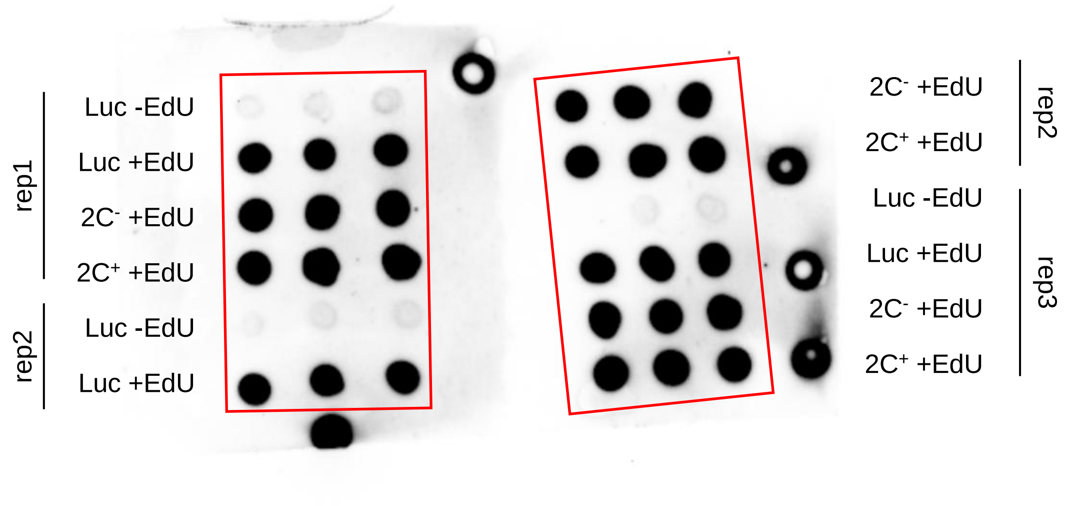

Anti-Vinculin

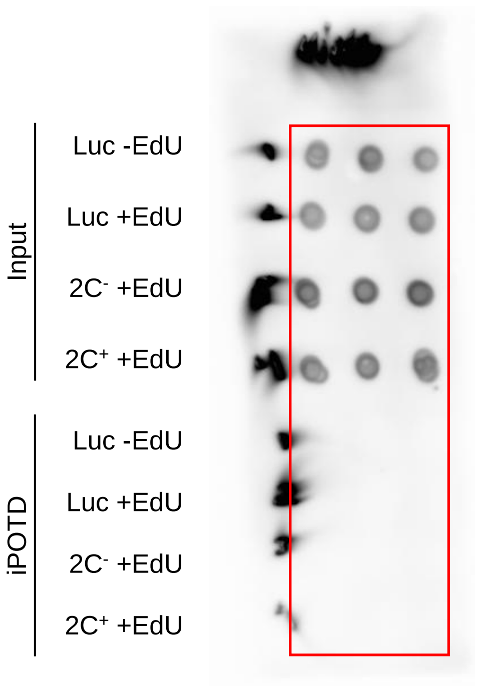

Figure 2-figure supplement 1, Source Data 2. Original membranes corresponding to Figure 2-figure supplement 1A. Independent DNA-mediated chromatin pull-down (iPOTD) eluates from sorted luciferase, 2C$^-$ and 2C$^+$ replicates in the absence or presence of EdU (±EdU) were analyzed by dot blot with an anti-H3 antibody (top). Input and eluates from equivalent preparations were incubated with an anti-vinculin antibody (bottom). Each condition was spotted in triplicates.
