# Supplementary material for: SMARCAD1 and TOPBP1 contribute to heterochromatin maintenance at the transition from the 2C-like to the pluripotent state
Source: eLife. 2025 Feb 19;12:RP87742. doi: 10.7554/eLife.87742 (PMC11839162; doi:10.7554/eLife.87742)
Supplement: Figure 3—figure supplement 1—source data 2. [file elife-87742-fig3-figsupp1-data2.zip › Figure 3-figure supplement 1_Source data 2/Figure 3-figure supplement 1_Source data 2.pdf]

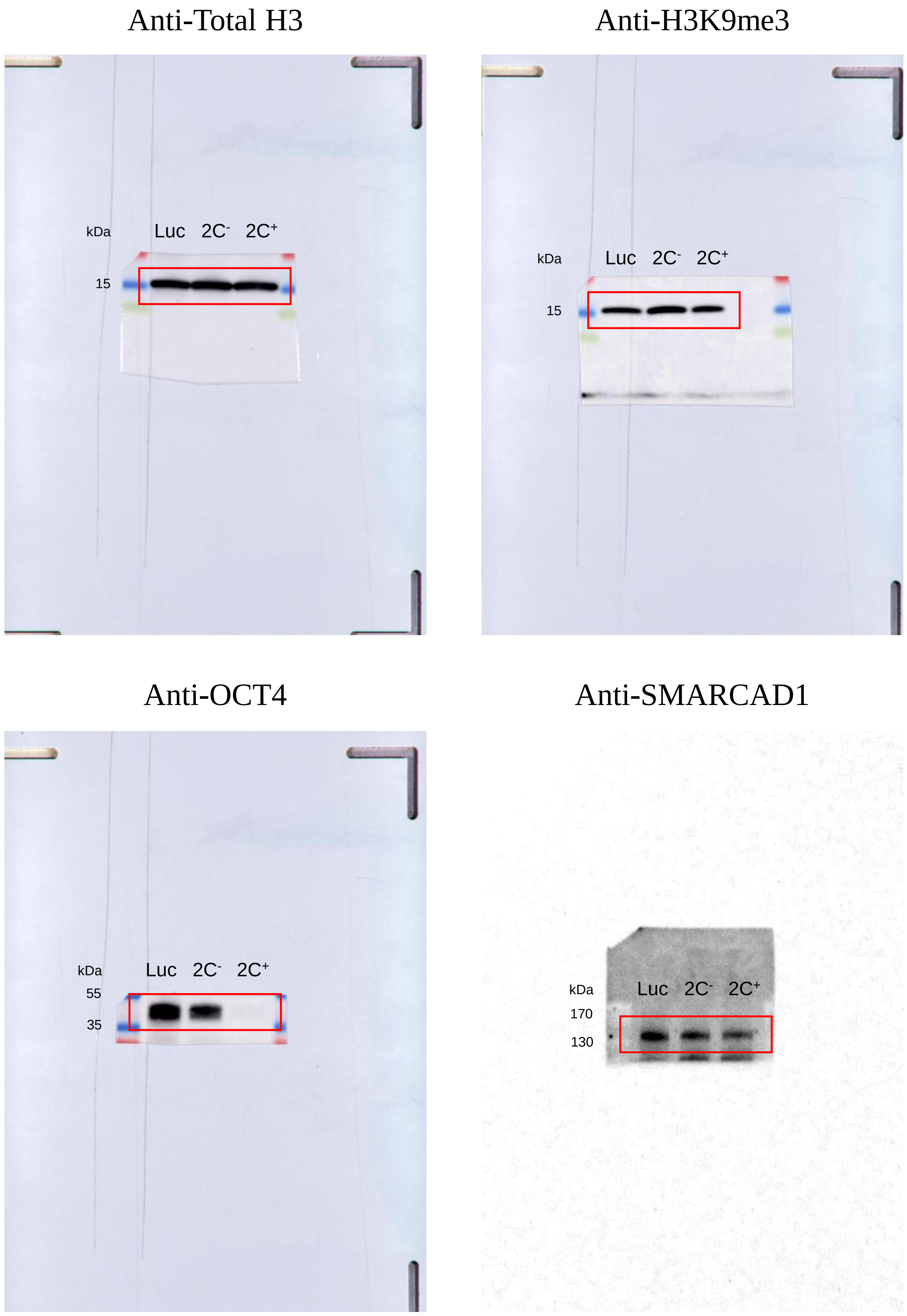

Figure 3-figure supplement 1, Source Data 2. Original membranes corresponding to Figure 3-figure supplement 1D. Representative Western Blots for luciferase (Luc), $2C^-$ and $2C^+$ cells. SMARCAD1, OCT4, H3K9me3 and total histone H3 blots are shown.
